# Distinguishing Target From Distractor in Stroop, Picture–Word, and Word–Word Interference Tasks

**DOI:** 10.3389/fpsyg.2015.01858

**Published:** 2015-12-15

**Authors:** Xenia Schmalz, Barbara Treccani, Claudio Mulatti

**Affiliations:** ^1^Dipartimento di Psicologia dello Sviluppo e della Socializzazione, Università degli Studi di Padova, Padova, Italy; ^2^ARC Centre of Excellence in Cognition and its Disorders, Macquarie University, Sydney, NSW, Australia; ^3^Dipartimento di Storia, Scienze dell’Uomo e della Formazione, Università degli Studi di Sassari, Sassari, Italy

**Keywords:** word–word interference, picture–word interference, Stroop test, lexical selection by competition, mental lexicon, selective attention

## Abstract

Lexical selection—both during reading aloud and speech production—involves selecting an intended word, while ignoring irrelevant lexical activation. This process has been studied by the use of interference tasks. Examples are the Stroop task, where participants ignore the written color word and name the color of the ink, picture–word interference tasks, where participants name a picture while ignoring a super-imposed written word, or word–word interference (WWI) tasks, where two words are presented and the participants need to respond to only one, based on an pre-determined visual feature (e.g., color, position). Here, we focus on the WWI task: it is theoretically impossible for existing models to explain how the cognitive system can respond to one stimulus and block the other, when they are presented by the same modality (i.e., they are both words). We describe a solution that can explain performance on the WWI task: drawing on the literature on visual attention, we propose that the system creates an object file for each perceived object, which is continuously updated with increasingly complete information about the stimulus, such as the task-relevant visual feature. Such a model can account for performance on all three tasks.

## Introduction

The cognitive system is often confronted with a set of stimuli, where one stimulus requires a response while others need to be ignored. This phenomenon is relevant to the process of lexical selection (Levelt et al., 1999): here, a target word needs to be produced, while irrelevant information (e.g., a semantically related word, or the word’s translation for multi-linguals) is ignored. This is only one step in the complex process of speech production, but it has been subject to some attention and controversy (e.g., Lupker, 1979; Finkbeiner and Caramazza, 2006; La Heij et al., 2006; Mahon et al., 2007).

Here, we consider whether existing models of lexical selection can adequately account for performance on three tasks that have been used to study the process of word selection in speech production: the Stroop task (Stroop, 1935; Klein, 1964; MacLeod, 1991), the picture–word interference (PWI) task (La Heij, 1988; Schriefers et al., 1990; Mahon et al., 2007), and the word–word interference (WWI) task (Glaser and Glaser, 1989; Waechter et al., 2011; Mulatti et al., 2015). These experimental tasks have in common the process of selecting a target, to which the participant needs to respond (e.g., by reading aloud, a lexical decision, or semantic categorization), and the need to ignore an irrelevant stimulus, the distractor. In the Stroop task the target is usually the font color and the distractor is the written color word, for the PWI task the target is a picture and the distractor a super-imposed written word, and for the WWI task the parti-cipants are presented with two words and need to respond to one based on a pre-determined characteristic (e.g., color, position).

A model of how the cognitive system performs selection should be able to explain performance on all three of these tasks. We argue that contemporary theories fail to account for performance on the WWI task, as it is theoretically impossible for the system in these models to ignore a distractor of the same type as a target (i.e., when both are words). We describe a model that can account for performance on all three tasks by creating a token, which combines, for each visual object, its identity with task-relevant visual features. We conclude with a brief discussion of how this model may account for phenomena in the more ecologically valid tasks of speech production and text reading.

In the current paper, we address an issue that arises in interference tasks: how does the system know which potentially activated lexical node belongs to the target, and which to the distractor? This is different—and logically preceding—from asking how relevant lexical entries are activated. The problem here is understanding how a given pattern of activation in memory is linked back to the stimulus evoking it. Ultimately, the task is to respond to only one of the two stimuli simultaneously presented, and so, the system needs to know that a given response corresponds to a given stimulus to decide what to process and what to gate.

## Previous Solutions

Any explanation of performance on the Stroop and PWI interference tasks relies on the concept of mental lexicons (Coltheart, 2004; but see Elman, 2004, 2009, 2011, for an alternative account of lexical knowledge). To explain the Stroop and PWI tasks, the mental lexicon needs to include three different domain-specific input modules: a color system (CS), a picture lexicon (PL), and an orthographic lexicon (OL). In addition, it needs a semantic system and a phonological output module. Each input module comprises a collection of domain-specific units, where each unit corresponds to a given element in that domain (e.g., each unit in the PL represents the structural description of an object), and is activated if that element is presented as input stimulus. Once a unit in one of the input modules is activated, it sends activation to the connected units in the semantic and phonological modules. In contrast to the units in the CS and PL, units in the OL also directly activate units in the phonological output lexicon, rather than only indirectly via the semantic system.

The existing proposals of performance on the PWI and Stroop are intrinsically linked to the notion of modality-specific input lexicons. These models achieve selective target activation based on a simple principle: in a PWI or Stroop task, the system needs to block the information from the wrong module. The system needs to monitor the activation in the input modules, because monitoring the activation in the later stages (i.e., the semantic system or phonological lexicon) would not provide the means to distinguish between information from different modes of input. Then, the system could deactivate the distractor activation, if it detects that it is sent from the distractor module which, in the case of the Stroop and PWI tasks, is the OL. Such deactivation could be achieved by disrupting processing of a stimulus that is provided by the “wrong” module.

Proposals along these lines have been made by several authors. Cohen et al. (1990) describe a parallel-distributed-processing computational model that can simulate results from Stroop-like tasks. Task instructions (ignore the written word vs. ignore the color of the font) are implemented as two input units that, via a set of hidden units, increase the activation for their respective target mode, and inhibit the stimulus provided by the distractor mode. WEAVER++ (Levelt et al., 1999), a leading computational model of word production, has been programmed to account for results on both Stroop and PWI tasks (Roelofs, 2003). Like the model of Cohen et al. (1990) the system tracks the input source of each stimulus: When activation spreads along the connections of the model’s network, it leaves activation tags at each node (Roelofs, 1993). These tags specify the source of the activation, and thus, in a PWI experiment, there are tags for both the picture stimulus and for the printed word stimulus: a response is selected only if its source tag corresponds to the picture.

These mechanisms rest on the same basic intuition, that pictures, colors, and printed words are inherently different. If the system can track the nature of a given item, it can distinguish targets from distractors. The identity of each stimulus does not influence these processes, since this would imply that the system knows the identity of the item before recognizing the item itself; instead, it only needs to classify the item in input as a member of the category of pictures (or colors, or printed words).

This family of explanations works when the two stimuli are processed through different input modules, but when the stimuli are of the same nature, it runs into fatal trouble. In a WWI task, participants are presented with two words simultaneously and are required to read one word while ignoring the other. Target and distractor can be distinguished because of their relative spatial position (La Heij et al., 1990; Mulatti et al., 2015), because of the different colors (Waechter et al., 2011), or their temporal order (Glaser and Glaser, 1989). Akin to the PWI task, evidence suggests that distractor affects target processing: unrelated low frequency distractors interfere more than unrelated high frequency distractor (Mulatti et al., 2015), target and distractor frequency exert additive effects on target processing (Mulatti et al., 2015), and semantically related distractors facilitate target processing (Waechter et al., 2011; Mulatti et al., 2015). This demonstrates that the distractors activate their orthographic and semantic representations to some extent. Therefore, accounting for the performance in the WWI task requires a mechanism that traces the source of the activation so that the system knows what has been activated by the distractor and what has been activated by the target. This mechanism, however, cannot be monitoring, tagging or biasing activation of a specific input module, because both stimuli in the WWI task are printed words, and activate nodes in the same module.

## An Alternative Approach

A model that could perform input control in the Stroop, PWI and WWI tasks would need to achieve the following: (1) at an early processing stage, it needs to assign the task-relevant visual feature to the stimulus, (2) the distractor is processed to some degree, and (3) when articulation occurs, the distractor has been suppressed (in the behavioral data, it is very rare for errors to occur, where the distractor is articulated instead of the target). Furthermore, to reflect psychologically valid mechanisms, the model should be applicable to all three tasks, as well as the extensive literature on visual attention and object recognition (Carr, 1999).

This problem has been described by Allport (1977), who stated, about Morton’s (1969) logogen model, that it “lacks a specific mechanism for relating particular logogen outputs to the particular stimuli that evoked them. In particular where more than one word, or nameable item, is presented at the same time, a mechanism is clearly required to integrate appropriately the nominal identities of the items—their logogen output—with their other physical attributes—location, color, size, etc.” (p. 525). Allport’s (1977) proposed solution is a mechanism which binds the word’s pre-categorical perceptual features with the word’s identity, or orthographic features, to form an episode. Once the task-dependent visual characteristics are linked to their respective orthographic information, the system knows which of the two lexical representations correspond to the target and which corresponds to the distractor, and the appropriate decision of what to read and what to ignore can be made. This approach is different from those explicitly proposed to account for interference tasks, because it does not require the tracking the input modes of each stimulus. Importantly, the idea of binding various attributed of the stimulus could be applied to explain how participants perform the WWI task as well as the PWI and Stroop tasks. In the following section, we describe a specified model based on Allport’s (1977) suggestion, and how it could account for performance on interference as well as reading tasks.

### Creating Proto-Words: Binding Visual Features

Upon stimulus presentation, the first step for the model is to detect that the display consists of two objects. In the WWI task, the system perceives the words as objects due to their visual distinctiveness compared to the background, and creates abstract representations for each of these objects. This lower-level selection process has been studied in great detail by researchers of visual attention. According to object file theories (e.g., Kahneman et al., 1992; Xu and Chun, 2009; Hayworth et al., 2011), a “file” is created for each object, which can be subsequently filled with continuously updated information about the object’s characteristics. At this stage, the objects have not yet been identified as words, but instead are organized bundles of the visual features of the word (“proto-words” in their terms).

### Orthographic Processing

As soon as proto-words are created, orthographic processing can be initiated, as two functionally independent sets of letter detectors—one for each proto-word—are constructed. After the creation of the letter sets, lexical processing can be initiated. The lexical processing stage creates a bottleneck, as only one word can undergo lexical processing at a given point in time (Coltheart et al., 2001). When the system is faced with multiple written words, it is assumed that the foveated word is prioritized (Engbert et al., 2005; Mulatti et al., 2015). This attentional gradient reflects the anatomy of the retina, where increasing distance from the fovea results in poorer spatial resolution. A further assumption of the model is that lexical processing is ballistic: once lexical processing of the item is initiated, it cannot be deactivated until identification has occurred.

During lexical processing, entries in the OL are activated, and this activation propagates—in an interactive and cascaded fashion—forward to the subsequent processing levels (Coltheart et al., 2001). The model posits the presence of an identification threshold in the orthographic input lexicon: as soon as this threshold is reached, the word can be treated as a tokenized instance of the type activation in the OL.

### Creating a Token: The Binding Visual and Orthographic Information and the Transfer to Verbal Working Memory

A token thus serves to bind the orthographic information to the specific instance of its occurrence, including the word’s non-orthographic characteristics. This process is based on the Simultaneous Type, Serial Token (TS^2^) model of Bowman and Wyble (2007). In the TS^2^ model, the token does not contain the information of the corresponding type: in the case of the WWI task, the token is created once an activation threshold in the OL is reached, meaning that subsequent cascaded processing is still required to activate semantic or phonological information. Thus, the token, rather than containing all of the information that is relevant for word production and semantic processing, acts as a pointer to where this information can be found. Subsequent processing is required to bind the newly created token to the activation in the phonological and semantic lexicons, as well as to its visual, pre-categorical representation. At this stage the system can continue processing that stimulus if it occupies the position of the target, or trigger deactivation if it occupies the position of the distractor. Once the relevant information associated with the token is bound, the task-relevant information is transferred to the phonological loop of working memory (Saito and Baddeley, 2004; Bowman and Wyble, 2007). From there, articulation of the target is initiated, and the correct response can be articulated.

## Beyond the WWI Task: Relevance of the Model to Other Settings

By using object files and tokens, the model described above proposes a mechanism by which the system can perform the WWI task. As we argue, it is theoretically impossible within existing proposals to account for the fact that the human participants are capable of ignoring a distractor while processing a target when these stem from the same source of input. Furthermore, the model allows for greater flexibility in incorporating visuo-attentional processes which may affect performance on interference tasks. This would provide a fruitful avenue for future research.

Future research is needed to establish how the model can account for performance on the PWI and Stroop tasks. Due to the similar nature of the three tasks, a mechanism explaining performance on one should be applicable to the other task, with relatively minor, task-specific modifications. The principle of creating object files and tokens could theoretically also work for the Stroop and PWI tasks. However, it would be a challenge for the model to create two files for a single visual object. In the case of the Stroop task, for example, the stimulus is a word written in a specific color, and the system needs to create a separate file for two aspects of the same stimulus. Beyond experimental scenarios, it is also worth considering whether the model could be applied to more naturalistic scenarios, and specifically, how it relates to word production and sentence reading.

### Interference Tasks and Word Production

The PWI interference task plays a central role in studying lexical selection in speech production (see Levelt et al., 1999, for a review). It is argued that the system, when translating a concept node to a phonological word form, needs to block competing word forms, thus posing a similar problem to the system as a PWI task. This view is not uncontroversial: it has been pointed out that in addition, the PWI task requires visuo-attentional, decision and selection processes that are not employed during speech production (Lupker, 1979; Carr, 1999; Finkbeiner and Caramazza, 2006). The degree to which the selection process involved in the PWI, WWI, and Stroop tasks—and in the model—reflects the selection process underlying lexical selection remains an open question. From a methodological perspective, a model which explains at least a proportion of the selection processes underlying the PWI task can help to isolate the task’s non-linguistic components from those that are directly related to the selection of a lexical node during speech production.

### Selecting Words During Reading

We argue that the WWI and the model in particular capture a cognitive mechanism that is particularly useful for text reading: namely, selecting a target word while ignoring the information provided by the surrounding words. Generally speaking, a well-specified model which incorporates such visuo-attentional mechanisms as well as higher-level orthographic processing can provide valuable insights and testable predictions about how these processes interact.

Text reading is generally studied with the use of eye-movement tracking. In the literature on reading and eye-movements, the degree to which all words in the visual field are processed is still under debate (e.g., Kliegl et al., 2006; Schotter et al., 2012; Angele et al., 2015). Several studies report when a word is fixated, the subsequent word influences its processing, especially when the fixated word is short. As in the WWI task (Mulatti et al., 2015), high frequency of the fixated and non-fixated words are facilitatory for target processing (Kennedy and Pynte, 2005; Kliegl et al., 2006) and have an additive effect (Schroyens et al., 1999; Kliegl et al., 2006). Future research could further explore the similarities between performance in the WWI task and in the task of text reading. Given a sufficiently high overlap in the underlying cognitive processing, the WWI could serve as an experimental task to study the processes underlying text reading.

## Conclusion

In summary, performance on the Stroop, PWI and WWI tasks reflects an important problem that is relevant to speech production and text reading. In all three tasks, information about the stimulus identity needs to be bound to the task-relevant visual information. We describe a specified model, based on a previous proposal by Allport (1977), that is capable of performing these tasks, and draws from literature of visual attention (Kahneman et al., 1992; Xu and Chun, 2009), object recognition (Bowman and Wyble, 2007; Hayworth et al., 2011), written word recognition (Coltheart et al., 2001), speech production (La Heij, 1988; La Heij et al., 1990; Levelt et al., 1999), and working memory (Saito and Baddeley, 2004).

### Conflict of Interest Statement

The authors declare that the research was conducted in the absence of any commercial or financial relationships that could be construed as a potential conflict of interest.
